# Host genetic diversity contributes to disease outcome in Crimean-Congo hemorrhagic fever virus infection

**DOI:** 10.1038/s44298-025-00100-5

**Published:** 2025-02-27

**Authors:** Deepashri Rao, Matthew Lewis, Kimberly Meade-White, Carl Shaia, Atsushi Okumura, Martin T. Ferris, Alexandra Schäfer, Ralph Baric, Heinz Feldmann, David W. Hawman

**Affiliations:** 1 Laboratory of Virology, Rocky Mountain Laboratories, NIAID/NIH, Hamilton, MT USA; 2 Rocky Mountain Veterinary Branch, Rocky Mountain Laboratories, NIAID/NIH, Hamilton, MT USA; 3 Department of Genetics, University of North Carolina at Chapel Hill, Chapel Hill, NC USA; 4 Department of Epidemiology, University of North Carolina at Chapel Hill, Chapel Hill, NC USA

**Keywords:** Virus-host interactions, Viral pathogenesis

## Abstract

The Crimean-Congo hemorrhagic fever virus (CCHFV) causes Crimean-Congo hemorrhagic fever (CCHF), a widely distributed disease with significant morbidity and mortality. The virus has high genetic diversity correlated with geographic distribution but limited temporal evolution within regions. Despite this, cases of CCHF within a region present as a spectrum of disease from often unrecognized asymptomatic infections to severe, fatal viral hemorrhagic fever, suggesting host factors may play a role in disease outcome. We investigated the effect of host genetic diversity on the outcome of CCHFV infection in the genetically diverse Collaborative cross (CC)-mouse model. Infected mice recapitulated the full spectrum of disease recognized in humans, and similar to human disease, virus replication, tissue pathology, and inflammatory responses were associated with disease severity. Our study demonstrates that host genetics contribute to disease outcome in CCHF infection and establishes the CC mouse resource as a model to understand how host genetic diversity contributes to CCHF outcome.

## Introduction

Crimean-Congo hemorrhagic fever (CCHF) is a severe, febrile illness that can progress to a viral hemorrhagic fever (VHF) in humans. The causative agent is the Crimean-Congo hemorrhagic fever virus (CCHFV), a tickborne RNA virus with a widespread geographic distribution^1,2^. Domestic and wild animals serve as amplifying hosts, while humans are incidental hosts. Exposure to the virus in humans primarily occurs through tick bites, as well as during handling of livestock during practices such as farming and butchering, which may lead to contact with infected blood^1,2^. Due to climate change, there is a possibility of an expansion in the geographic range of the tick vector^3^, resulting in a higher risk of more human exposure to the virus. Case fatality rate is variable but can be higher than 30% in endemic regions^4^.

Interestingly, although CCHFV can productively infect a wide range of domestic and wild animal species, with studies showing seroconversion in rabbits, cattle, and even tortoises^5,6^ symptomatic disease has only been reported in humans. Additionally, within humans, infection with CCHFV results in a spectrum of disease, from asymptomatic infection, to severe, sometimes lethal disease^7,8^. The virus is also genetically diverse, with diversity correlating with geography^9,10^. However, within a geographical region, there is often minimal diversity of the virus, with studies showing strong sequence conservation in strains of CCHFV isolated from the same regions decades apart^11,12^. It is, therefore, likely that within a region, people are infected with similar strains of the virus and yet exhibit different outcomes. A number of factors, including viral determinants, virus dose, route of exposure, host immune responses, and access to healthcare resources, are likely to contribute to disease outcome and case fatality rates. Several studies have demonstrated the association of polymorphisms in certain innate signaling genes to CCHF disease severity in humans, suggesting that host genetics may also contribute to disease outcome^13–18^. However, the effect of genetic diversity on CCHF disease is poorly understood, and the study of innate immunity to CCHFV is limited by the historical requirement that mice be deficient in type I interferon to develop symptomatic disease.

We recently developed a mouse-adapted strain of CCHFV (MA-CCHFV) that infects and results in symptomatic disease in immunocompetent mice^19^. With this model, we also identified sex-linked differences in disease severity, with male mice developing more severe disease than female mice. These sex-linked differences were maintained across several strains of mice tested, including C57BL/6 J, C57BL/6NCR, and BALB/c/J mice. Interestingly, both male and female 129S1 mice were largely resistant to disease^19^. These findings suggested that with the MA-CCHFV virus, there exist sex and host strain-specific differences in disease outcome.

Since these conventional inbred strains represent only a subset of mouse genetic diversity, here we explored the effect of host genetics on disease progression after infection with the MA-CCHFV using the genetically diverse Collaborative Cross (CC) mouse^20–24^, a genetic reference population. Infection of male and female mice of ten CC strains resulted in a wide spectrum of disease outcomes, from asymptomatic infection to lethal disease. Disease severity in CC mice showed similar correlates to disease severity in human CCHF cases, such as viral replication, liver pathology, and inflammatory cytokine production. Our findings demonstrate that host genetics contribute to disease outcomes in MA-CCHFV infection of mice and establish the CC mouse resource for continued study of how host responses contribute to CCHF disease outcomes.

## Methods

### Biosafety and ethics

All infectious work with CCHFV was performed in the biosafety level 4 (BSL-4) maximum containment laboratory at the Integrated Research Facility, Rocky Mountain Laboratories (RML), Division of Intramural Research (DIR), National Institute of Allergy and Infectious Diseases (NIAID), National Institutes of Health (NIH) according to standard operating procedures (SOPs) approved by the RML Institutional Biosafety Committee (IBC). All animal studies were approved by the RML Institutional Animal Care and Use Committee (IACUC) and performed according to the guidelines of the Association for Assessment and Accreditation of Laboratory Animal Care, International, and the Office of Laboratory Animal Welfare by trained and experienced personnel. Humane endpoint criteria in compliance with IACUC-approved scoring parameters were used to determine when animals should be humanely euthanized.

### Mice

Male and female CC mice, 7–15 weeks of age and bred at the University of North Carolina at Chapel Hill’s Systems Genetics Core Facility, purchased between January and October 2023, were used in the study. Mice from ten different CC strains (CC003/Unc, CC046/Unc, CC080/TauUnc, CC004/TauUnc, CC037/TauUnc, CC002/Unc, CC051/TauUnc, CC037/TauUnc, CC030/GeniUnc, CC042/GeniUnc and CC012/GeniUnc, hereafter referred to without suffixes) were included. Mice were acclimatized to ABSL-4 conditions prior to the start of experiments and were provided with food, water, and nesting material ad libitum. Cage changes were performed by animal caretakers every 14 days. Mice were randomly assigned to groups. Only the histology team was blinded to the study. For procedures requiring anesthesia, animals were anesthetized using isoflurane. Following infection, mice were weighed daily up to day 14 or euthanasia timepoint, and surviving mouse weights were recorded every 3 days until the study endpoint. For survival studies, anesthetized mice were implanted with telemetry transponders (UCT-2112, UID) via subcutaneous implantation to enable monitoring of body temperature and activity levels. Implanted mice were allowed to recover for at least one week prior to CCHFV challenge. Data were recorded continuously with a zone interval of 250 ms, 2 cycles per series, and a 1 s series delay. Data are reported as the mean of readings collected during 6-h intervals corresponding to vivarium light–dark cycles. Following survival studies, the disease was further characterized in a second set of mice of 6 CC strains. Infected mice were euthanized at peak disease (corresponding with the highest weight loss in survival studies) for virological, immunological, and histopathology analyses. Mice were euthanized using exsanguination under deep anesthesia followed by cervical dislocation or isoflurane overdose followed by cervical dislocation.

### Virus stock and infections

The stock of MA-CCHFV used here is the same as described previously^25^. Anesthetized mice were inoculated intraperitoneally with 10^4^ TCID_50_ of MA-CCHFV in 100 μL of Dulbecco’s Modified Eagle Medium (DMEM) or mock infected with DMEM only.

### qRT-PCR

Viral RNA copies in the blood, liver, and spleen were quantified by qRT-PCR as previously described^19^. Limit of detection (LoD) of the assay was determined by extrapolating the standard curve to the copy number given by a Ct (crossing of threshold) value of 40. Samples without any amplification were set at the LoD. The last standard to amplify was set as the limit of quantification (LoQ) of the assay.

### Cytokine analysis

The levels of cytokines in the sera were analyzed by the Bio-Plex Pro Mouse Cytokine 23-plex Assay (Bio-Rad, cat #M60009RDPD) according to manufacturer’s instructions.

### Enzyme-linked immunosorbent assay (ELISA)

IgG responses to CCHFV in the sera were evaluated by an in-house ELISA as previously described^26^. Absorbance of negative samples were used to determine limits of detection.

### Histology and immunohistochemistry

Liver and spleen tissues were harvested from mice at peak disease for each strain. Tissues were fixed in 10% Neutral Buffered Formalin x2 changes, for a minimum of 7 days. Fixed tissues were placed in cassettes and processed with a Sakura VIP-6 Tissue Tek, on a 12-hour automated schedule, using a graded series of ethanol, xylene, and PureAffin. Embedded tissues were sectioned at 5μm and dried overnight at 42°C prior to staining. Specific anti-CCHFV immunoreactivity was detected using Rabbit anti-CCHFV N (IBT Bioservices, cat #04-0011) at a 1:2000 dilution. The secondary antibody was from the Immpress-VR horse anti-rabbit IgG polymer kit (Vector Laboratories, cat #MP-6401). The tissues were then processed for immunohistochemistry using the Discovery Ultra automated stainer (Ventana Medical Systems) with a ChromoMap DAB kit (Roche Tissue Diagnostics, cat#760-159). Necrosis was scored on a scale of 0 to 5, with 0 representing no necrosis, 1 representing minimal foci (1–10% of the tissue section), 2 mild (11–25%), 3 moderate (26–50%), 4 marked (51–75%) and 5 severe (76–100% of the tissue affected). Anti-CCHFV immunoreactivity was also scored similarly on a scale of 0 to 5, with 0 representing no immunoreactivity, 1 representing minimal immunoreactivity (1–10% of the tissue section), 2 mild (11–25%), 3 moderate (26–50%), 4 marked (51–75%) and 5 high immunoreactivity (76–100% of the tissue affected).

### Statistics

Statistics were performed on GraphPad Prism 10. Comparison of viral loads, inflammatory cytokine levels, and IgG levels was performed using one-way ANOVA with Tukey’s multiple comparison test. Weight loss between male and female mice within each strain was compared using two-way ANOVA or mixed-effect analysis with Sidak’s multiple comparison test. Sample sizes were determined based on previous experience with the MA-CCHFV model, and mice were assigned to groups in a random manner. For survival studies, 5 male and 5 five female mice of each strain were analyzed for strains CC003, CC042, CC030, CC051, CC080, and CC002. For strain CC046, 10 mice per sex, and for strains CC004 and CC012, 9 mice per sex were analyzed (1 male mouse from CC004 and CC012 did not recover from anesthesia, and 1 female mouse of each of these strains was euthanized for non-study related reasons). Strain CC037 had 5 males, and 3 females as one female mouse did not recover from anesthesia, and another one was euthanized for non-study related reasons. For timed necropsy studies, 6 mice per sex per strain were included for MA-CCHFV-infected groups, and 2 mice per sex per strain were included for mock-infected groups.

## Results

### Infection with MA-CCHFV results in a spectrum of clinical manifestations in CC mice

**Fig. 1:**
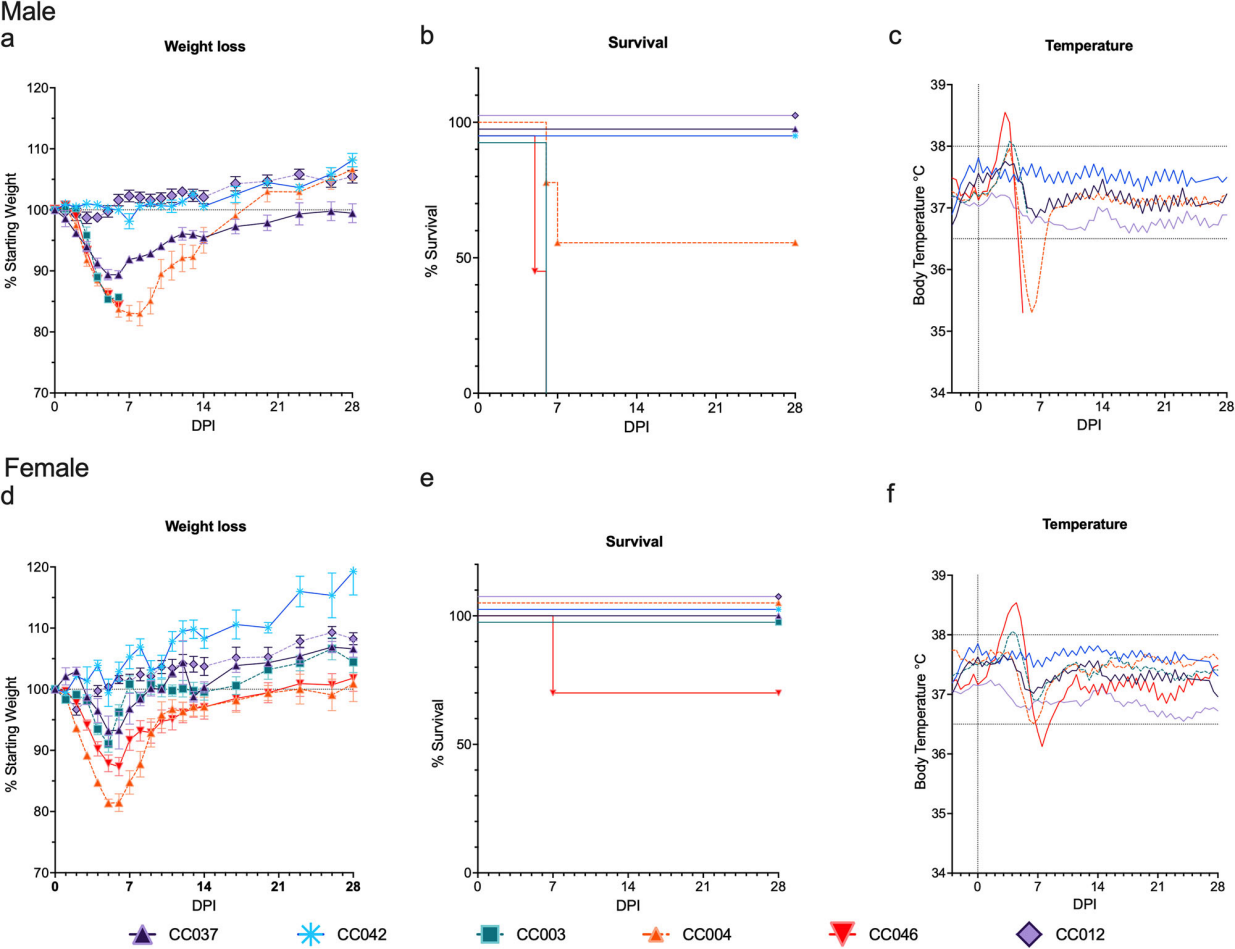
Infection of CC mice with MA-CCHFV results in a spectrum of disease. Male (**a**–**d**) or female (**e**–**h**) mice of six CC strains were infected with 10^4^ TCID_50_ of MA-CCHFV intraperitoneally and monitored for clinical signs and symptoms of disease. *N* = 5 mice per sex for strains CC003 and CC042. *N* = 5 males and three females for strain CC037. *N* = 9 mice per sex for strains CC004 and CC012. *N* = 10 mice per sex for strain CC046. Mice were weighed daily, (**a**, **d**), monitored for survival (**b**, **e**) and body temperatures (**c**, **f**) were measured by the cage telemetry system. Data are shown as the mean plus standard error of the mean (SEM) (**a**, **d**). Experiment was performed once for all strains, except CC046, CC012, and CC004, for which survival studies were performed twice. For these three strains, the weight loss and survival data are combined from the two independent experiments.

Since infection with MA-CCHFV results in disease in strains of immunocompetent conventional inbred WT mice with minimal genetic diversity^19^, we hypothesized that infection of the genetically diverse CC mice would result in a range of clinical presentations across these genotypes. Male and female mice of 10 CC strains were infected with MA-CCHFV and monitored for the development of clinical signs. Mice were categorized into groups based on peak body weight loss and the presence or absence of mortality into 100% lethal (100% mortality), 25, 45, or 60% lethal, moderate (16–25% weight loss with no mortality), mild (5–15% weight loss with no mortality), and asymptomatic (resistant to clinical disease) (0–4% weight loss with no mortality) (Table 1). Infection of male CC mice resulted in a range of disease outcomes, from severe, lethal disease to moderate disease and mild and asymptomatic infections, as indicated by weight loss, fluctuations in body temperature, reduced activity levels, and reduced survival (Fig. 1a–c, Supplementary Fig. 1a and 2a–d). The infection was uniformly lethal in male mice of two strains, CC003 and CC046, both of which rapidly lost weight, exhibited hyperthermia and lethargy and all succumbed to the disease at 5–6 days post infection (dpi) (Fig. 1a, b, Supplementary Fig. 1a). Male mice of strains CC080 and CC004 also had lethality, with 60% mortality in CC080 mice by 6 dpi (3/5 mice succumbed) (Supplementary Fig. 2b) and 44.4% in CC004 mice, with mice succumbing on 6–7 dpi (4/9 mice succumbed) (Fig. 1b). Both strains developed hyperthermia followed by hypothermia corresponding to peak weight loss, along with a decline in activity levels, before reaching euthanasia criteria or recovering (Fig. 1a, c, Supplementary Fig. 1a, Supplementary Fig. 2a, c, d). Strains CC037, CC002, and CC051 showed mild disease in male mice with a peak weight loss of 8–11% of their starting body weight and 100% survival (Fig. 1a, b, Supplementary Fig. 2a, b). Strain CC037 also displayed a biphasic disease pattern, with peak weight loss at day 5, and subsequently gaining weight until day 14, when body weight again dropped slightly before complete recovery (Fig. 1a). All three strains showed slight elevations in body temperatures and a slight reduction in activity levels coinciding with peak weight loss (Fig. 1c, Supplementary Figs. 1a and 2c, d). Male mice of strain CC030 also displayed mild disease with 8% body weight loss rapidly following infection on day 1, before quickly recovering the lost weight by day 3 and all survived (Supplementary Fig. 2a, b). There were no changes in body temperature or activity levels of these mice throughout the course of infection. Two strains, CC042 and CC012 did not show any weight loss, changes in body temperature or otherwise overt clinical disease. Strain CC012 showed mildly reduced activity levels during the first week of infection, but subsequently returned to normal activity levels (Supplementary Fig. 1a).

**Table 1:** Categorization of mice into groups based on body weight loss and mortality

Sex	Strain	Body weight loss at peak disease (%)	Mortality (%)	Category
Male	CC003	15	100	100% lethality
	CC046	15	100	100% lethality
	CC004	17	44.4	~45% lethality
	CC037	11	0	Mild
	CC042	0	0	Resistant–asymptomatic
	CC012	0	0	Resistant–asymptomatic
	CC030	8	0	Mild
	CC051	8	0	Mild
	CC080	13	60	60% lethality
	CC002	8	0	Mild
Female	CC003	10	0	Mild
	CC046	10	30	30% lethality
	CC004	20	0	Moderate
	CC037	5	0	Mild
	CC042	0	0	Resistant–asymptomatic
	CC012	4	0	Resistant–asymptomatic
	CC030	8	0	Mild
	CC051	6	0	Mild
	CC080	5	0	Mild
	CC002	5	0	Mild

Female mice infected with MA-CCHFV also displayed a spectrum ranging from asymptomatic to moderate disease (Fig. 1d and Supplementary Fig. 2e). Strain CC046, in which male mice had 100% lethality, had 30% lethality (3/10 mice succumbed) in females (Fig. 1e). Female CC046 exhibited hyper- followed by hypothermia (Fig. 1f) and there was a prolonged recovery with mice not returning to starting weight until 21 dpi (Fig. 1d). However, body temperature (Fig. 1f) and activity levels (Supplementary Fig. 1b) returned to baseline by 14 dpi. Lethal disease was not observed in female mice of any of the other 9 strains tested, (Fig. 1e and Supplementary Fig. 2f)) suggesting sex-linked differences observed in inbred laboratory strains of mice remain in genetically diverse, but inbred CC mice. Female mice of strain CC004 showed moderate disease with 20% weight loss at peak disease on day 5. Coinciding with peak weight loss, mice exhibited a decline in body temperature and activity levels before recovery. A majority of the strains tested (6/10) only developed mild disease in female mice, with <10% weight loss and mild disturbance to temperatures (Fig. 1d–f). Female mice of the two strains that did not show any clinical symptoms of disease in males, CC042 and CC012, were also resistant to disease, with little-to-no weight loss or changes in body temperature or activity levels observed (Fig. 1d–f).

Since the MA-CCHFV strain displays sex-linked differences in disease severity, with male mice developing higher weight loss than female mice in several strains tested^19^, we also compared weight loss between male and female mice of each CC line that showed weight loss (8/10 strains) (Supplementary Fig. 3). The same pattern of higher weight loss in male mice than female mice was observed in 6 of the 8 strains: CC037, CC046, CC051, CC080, CC002, and CC003. The weight loss pattern was similar in male and female mice of strain CC030, both of which had mild weight loss early post-infection before complete recovery (Supplementary Fig. 3h). Interestingly, female CC004 mice presented with slightly higher weight loss than male mice (Supplementary Fig. 3g), despite the 45% lethality observed in male mice (Fig. 1b) and 100% survival in female mice (Fig. 1e).

Together, these findings demonstrate that the CC mice recapitulate the full spectrum of CCHF disease observed in humans. Further the sex-bias in disease severity with MA-CCHFV infection of in-bred laboratory strains of mice such as C57BL6/J^19^ was largely maintained in all CC strains tested. The notable exception were the strain CC004 mice which showed greater weight loss in female mice but greater lethality in male mice.

### MA-CCHFV replicates in all CC strains tested

In humans, CCHF outcome correlates with viral load, organ damage, and inflammatory immune responses^27–32^. Therefore, we performed a more in-depth characterization of CCHFV infection in a subset of CC mice to correlate overt disease outcomes with virology, tissue pathology, and inflammation. Based on the survival studies, we selected 6 strains for further characterization of CCHFV infection: CC003 (♂: 100% mortality, ♀: mild disease), CC046 (♂: 100% mortality, ♀: 30% mortality), CC004 (♂: 45% mortality, ♀: > weight loss than ♂) CC037 (♂ and ♀: mild disease), and strains CC012 and CC042 (♂ and ♀: no apparent disease).

**Fig. 2:**
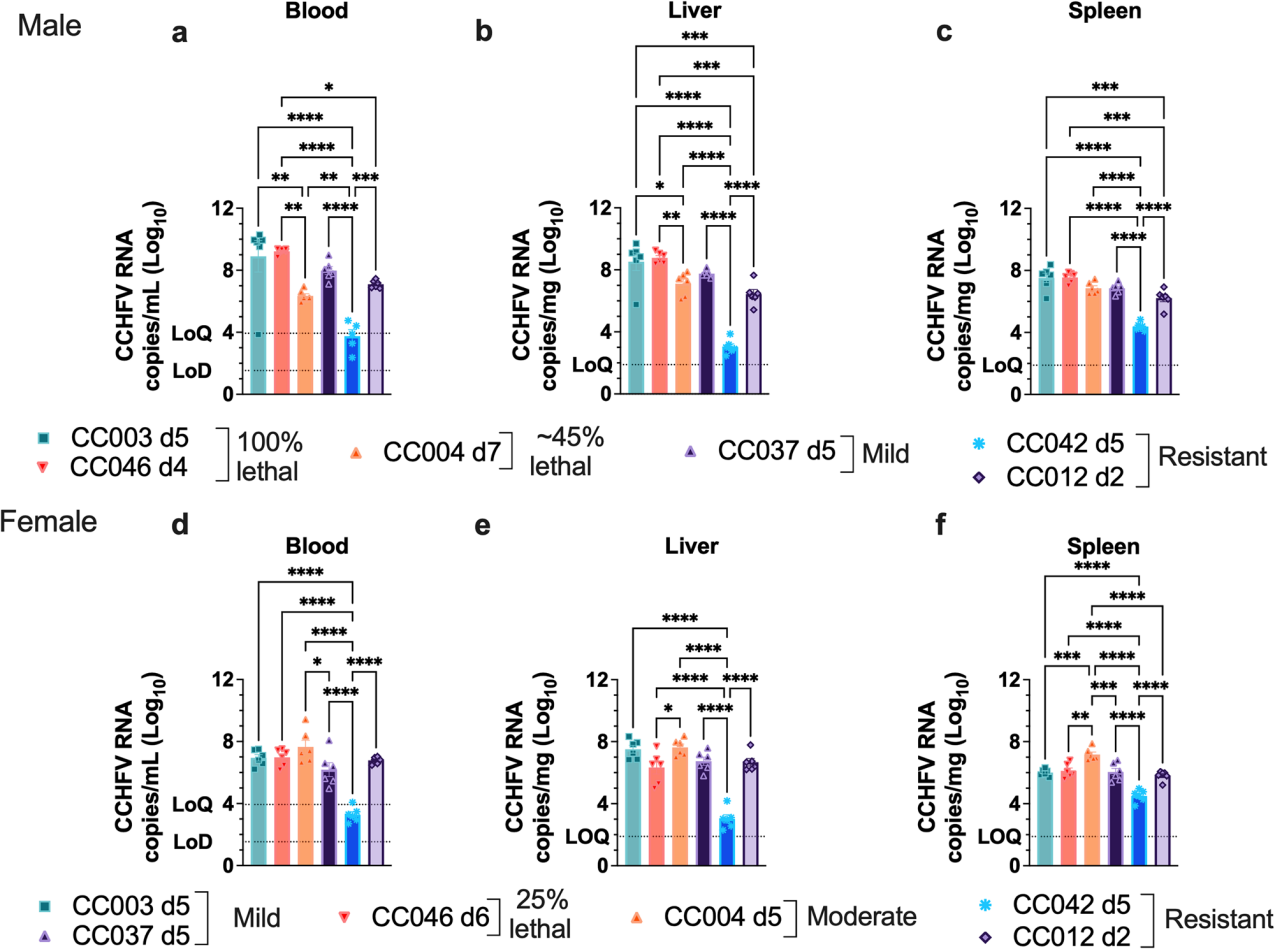
Disease progression in CC mice following MA-CCHFV infection is associated with virus replication. Groups of male (**a**–**c**) or female (**d**–**f**) mice of 6 CC strains were infected with 10^4^ TCID_50_ of MA-CCHFV intraperitoneally and euthanized at peak disease for each sex of each strain. *N* = 6 mice per sex per strain. CCHFV RNA loads in the indicated tissues were evaluated by qRT-PCR. Data presented as mean plus SEM. Statistics were calculated with a one-way ANOVA with Tukey’s multiple comparison test for comparisons. *P < 0.05, **P < 0.01, ***P < 0.001, ****P < 0.0001.

Mice were infected with MA-CCHFV on day 0 and euthanized at peak disease, corresponding with the highest weight loss for each sex of each strain in the survival study. Analysis of viral RNA loads showed that both male and female mice of all 6 CC strains tested had detectable viremia (Fig. 2a, d) and viral RNA in the livers (Fig. 2b, e) and spleens (Fig. 2c, f) at peak disease. Mice that were resistant to clinical disease largely had significantly reduced viral loads compared to mice exhibiting more severe disease (Fig. 2a–f). In contrast, female CC012 mice, which were resistant to clinical disease, had similar viral loads to mice that developed more severe disease (Fig. 2d–f). However, the day of necropsy was timed to peak clinical disease and thus varied across each strain. We cannot exclude the possibility that these differences observed may be due to differences in the day of sample collection. We also confirmed productive infection in CC042 and CC012 by analyzing serum IgG responses to CCHFV in these two strains at 28 dpi. CCHFV-specific IgG was observed in both sexes of CC012 and CC042 (Supplementary Fig. 4). Overall, these results suggest that resistance to disease upon infection with CCHFV as observed in strains CC012 and CC042, is not due to an inherent inability of these two strains to be infected. Instead, MA-CCHFV replicated in all CC mouse strains tested.

### MA-CCHFV causes liver pathology associated with disease severity in CC mice

**Fig. 3:**
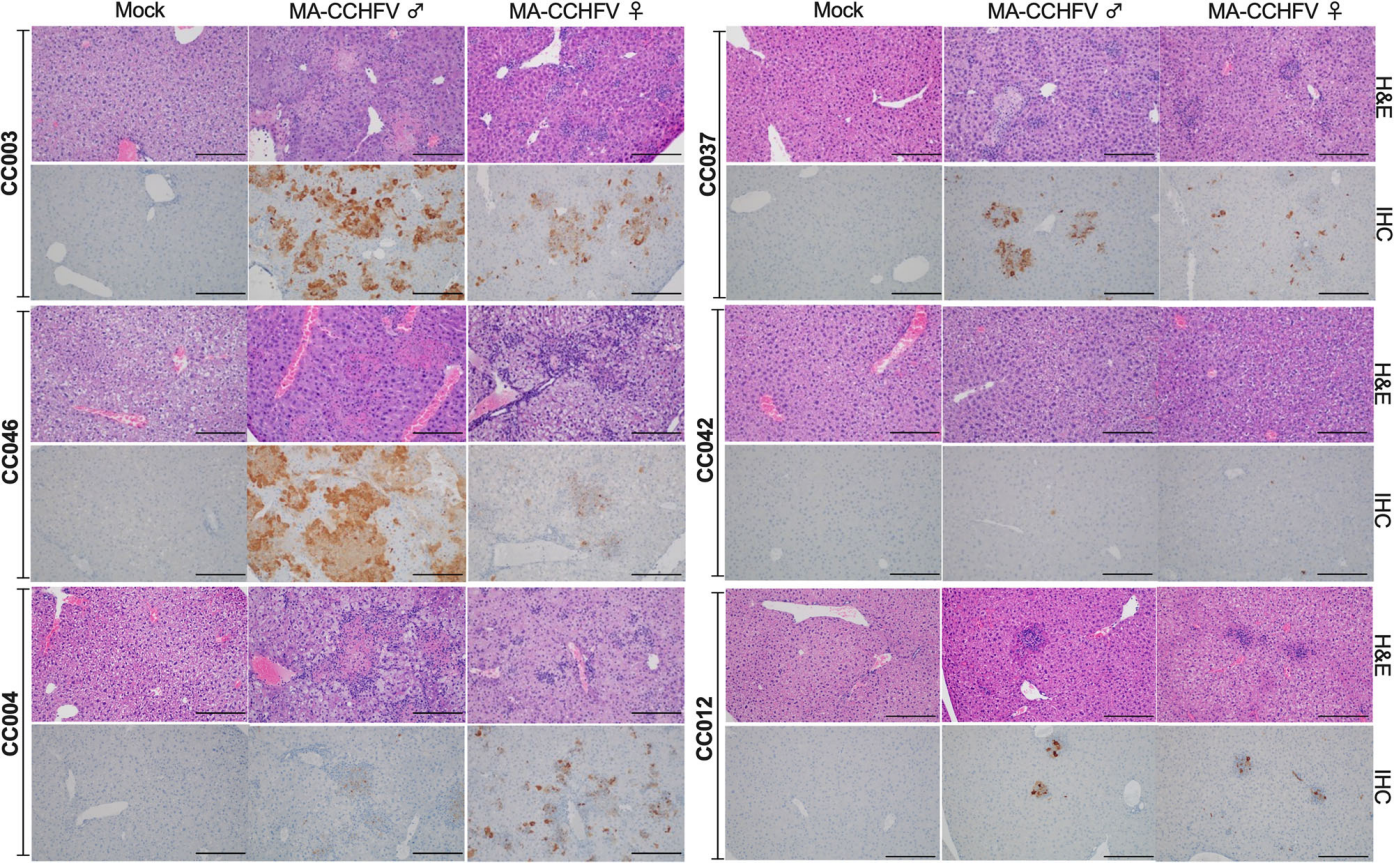
CC mice infected with MA-CCHFV develop liver pathology correlating with disease severity. Groups of male and female mice of 6 CC strains were infected with 10^4^ TCID_50_ of MA-CCHFV via the intraperitoneal route or mock-infected. *N* = 6 mice per sex per strain for CCHFV-infected groups. *N* = 2 mice per sex per strain for mock-infected groups, except strain CC037, for which *N* = 1 male and 1 female mouse for mock-infected group. At peak disease, mice were euthanized, and tissues were fixed in formalin and paraffin-embedded sections stained with H and E or an antibody against the CCHFV NP to identify viral antigens (IHC). Representative images of liver are shown. Images are shown at ×200 magnification, and the scale bar indicates 100 μm. Study performed once.

Similar to CCHFV infection in humans, the liver is the primary target of MA-CCHFV, which causes significant pathology upon infection of WT mice^19^. Next, we examined histological evidence of liver pathology in the 6 CC strains infected with MA-CCHFV. Overall, pathology in the liver and spleen correlated with disease severity. CC mice that developed more severe disease and had higher viral loads (CC003, CC046, CC004) had greater pathological findings than strains with milder (CC0037) or no disease (CC042, CC012). This correlation to disease severity was also seen when comparing disease severity and pathology between the sexes of each strain (Fig. 3, Supplementary Figs. 5 and 6). Collectively, these findings demonstrate the MA-CCHFV infection of CC mice results in pathology of the liver and spleen that correlates with disease severity across genetic backgrounds.

### MA-CCHFV induces an inflammatory cytokine response associated with disease severity in CC mice

**Fig. 4:**
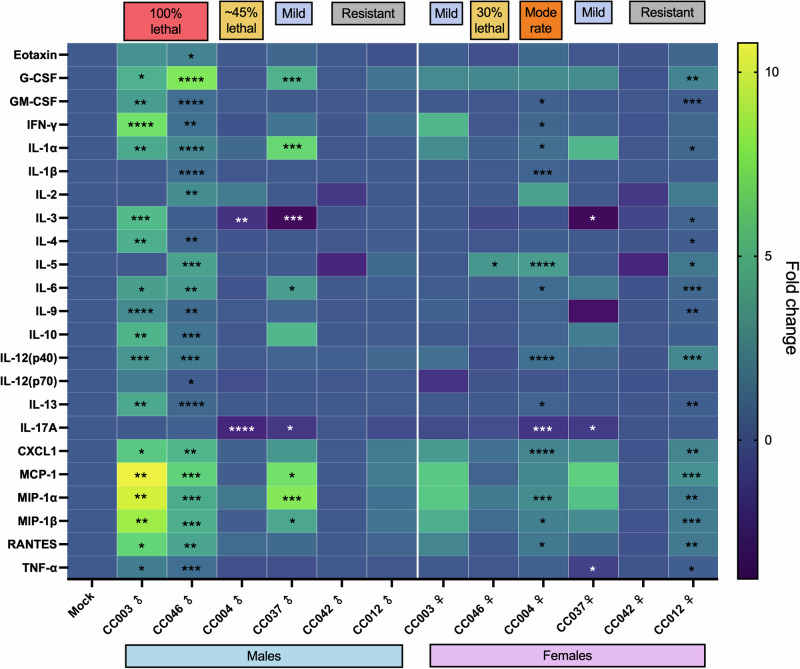
An inflammatory immune response associated with disease severity develops in MA-CCHFV-infected CC mice. Groups of male and female CC mice were infected with 10^4^ TCID_50_ of MA-CCHFV via the intraperitoneal route or mock-infected. *N* = 6 mice per sex per strain for CCHFV-infected groups. *N* = 2 mice per sex per strain for mock-infected groups, except strain CC037, for which *N* = 1 male and 1 female mouse for the mock-infected group. At peak disease, cytokine levels in the sera were measured by 23-plex cytokine assay. Data showed a fold change in the levels of each cytokine in the MA-CCHFV-infected mice over mocks. Study was performed once. Statistics were calculated with a one-way ANOVA with Tukey’s multiple comparison test. Asterisks indicate significantly higher (in black), or lower (in white) levels of cytokines in male and female MA-CCHFV-infected mice compared to mocks- *P < 0.05, **P < 0.01, ***P < 0.001, ****P < 0.0001.

Disease severity in humans and MA-CCHFV infection of WT mice correlates with systemic production of multiple inflammatory cytokines^19,30,32,33^. Therefore, we evaluated serum cytokine responses in male and female mice of the 6 CC strains at peak disease. Compared to mock-infected mice, the 100% lethal disease in male mice of strains CC003 and CC046 was associated with significantly higher levels of multiple cytokines, including Eotaxin, G-CSF, GM-CSF, IFN-γ, IL-1α, IL-4, IL-6, IL-9. IL-10, IL-12(p40), IL-13, CXCL1, MCP-1, MIP-1α, MIP-1β, RANTES, and TNFα (Fig. 4). Female mice of both strains did not have significant elevations in most of the cytokines tested, except IL-5 in CC046, despite the 30% mortality observed in these mice. Interestingly, strain CC004, in which the infection was lethal in ~40% of male mice, did not have increases in any cytokines over mock mice. In contrast, the higher weight loss in female mice of this strain was associated with significantly higher levels of several inflammatory cytokines in the serum at peak disease (day 5 post-infection) compared to mocks. Both male and female CC042 mice, which showed no disease, had no changes to serum cytokine levels, while similarly, male CC012 mice also had no changes to cytokine levels. Female CC012 mice, which had slight weight loss, had slightly but significantly increased levels of several cytokines. The individual cytokine profiles for each strain and sex are presented in Supplementary Fig. 7–12. Overall, lethal disease was associated with increased inflammatory cytokines such as Eotaxin, G-CSF, GM-CSF, IFN-γ, IL-1α, IL-4, IL-6, IL-9, IL-10, IL-12(p40), IL-13, CXCL1, MCP-1, MIP-1α, MIP-1β, RANTES and TNFα, several of which have been associated with fatal human cases of CCHF^31–33^. However, mortality in male CC004 mice or female CC046 mice was associated with little change in cytokine levels. Taken together, these results suggest that disease severity in CC mice correlates with inflammatory cytokine production, but other disease processes may also contribute.

## Discussion

For many VHFs, host immunopathology contributes significantly to morbidity and mortality but for CCHF it remains largely unclear how host factors contribute to disease outcome. Although results from some studies indicate that genetic variation in humans, such as polymorphisms in NF-κB, TLRs, and IFNα, may determine the severity of CCHF disease^13–18^, this has not been characterized in depth nor modeled in genetically diverse systems. Investigations of host innate responses to CCHFV have also been limited by the requirement for mice to be deficient in type I IFN signaling to model human disease. Thus, a mechanistic understanding of the contribution of host genetics to CCHF disease is lacking. This report demonstrates that host genetic diversity contributes to CCHF disease outcome. We observed the full spectrum of CCHF disease reported in humans in CC strains infected with MA-CCHFV, sharing similar correlates of outcome such as viral load, tissue pathology, and inflammatory responses as human disease. Thus our work establishes a tractable mouse model system to study host determinants of CCHF outcome. The strains developing lethal disease represent a useful model for identifying host genes that could potentially direct the course of CCHFV infection while also providing a severe disease model for evaluating interventions such as antivirals and vaccines.

A majority of the CC strains screened presented with mild to moderate disease, and two strains were resistant to clinical disease in both male and female mice. This is consistent with human infections, a majority of which are likely subclinical and underappreciated^34,35^. Our data indicate that asymptomatic or subclinical infections were not due to resistance to infection but instead likely due to host responses that resulted in rapid control of the infection with minimal immunopathology. This data is consistent with serological evidence indicating productive infection of numerous animal species in the absence of clinical disease^5^, suggesting that although CCHFV can infect numerous animal species, unique determinants within the human host enable symptomatic disease. Interestingly, we also measured distinct cytokine responses between mild and asymptomatic disease, suggesting that distinct cytokine responses and likely overall host immune responses to the infection exist in asymptomatic, mild, and severe CCHFV infections. These models are useful for understanding protective host responses to CCHFV infection and could be utilized to investigate host-directed therapeutics that promote protective host responses while limiting those that lead to immunopathology. We were also able to identify more subtle disease phenotypes, such as mice with mild weight loss but prolonged recovery (CC051 males) versus mice with rapid severe weight loss but rapid recovery (CC080 males). These models may be useful for studying the long-term sequelae that may follow recovery from acute CCHF, an area in need of further research.

Cumulatively, our data highlight the utility of the CC mice to model the variable disease reported in CCHFV-infected humans. The CC mice have proven valuable in the study of other viruses, including Ebola in CC and CC recombinant inbred intercrossed (CC-RIX) mice using a mouse-adapted strain of Ebola virus (MA-EBOV)^21,22,36^. More recently, strains CC051 and CC004 were investigated for susceptibility to MA-EBOV infection^22^. Infection of CC051 showed mild to moderate disease, and CC004 was highly susceptible to EBOV infection and showed uniform mortality. Thus, MA-EBOV infection in these two strains was more severe than MA-CCHFV infection, suggesting that genes involved in the susceptibility to diverse VHF viruses may be distinct.

The sex-linked bias observed with infection of inbred laboratory mouse strains with MA-CCHFV was largely maintained in six out of the eight CC strains that showed disease, suggesting the mechanism is conserved in the genetically diverse CC model. However, we identified one strain, CC004, in which female mice showed higher weight loss, increased viral loads, and inflammatory cytokines than male mice at peak disease. Sex differences in disease presentations have been described in the CC for other infectious diseases^37,38^. Our finding suggests a role for host genetics in driving sex differences in the immune response to MA-CCHFV infection. MA-CCHFV infection of CC mice provides an opportunity to investigate host genes associated with sex-linked determinants of the outcome of viral infections, an understudied area in the context of VHFs. We have provided a summary of the clinical manifestations of MA-CCHFV infection in the six CC strains and the inbred C57BL/6 strain in Table 2.

**Table 2:** Overview of the clinical manifestations of MA-CCHFV infection in inbred C57BL/6 and genetically diverse CC mice

Strain	MA-CCHFV dose	Males–salient clinical symptoms	Females–salient clinical symptoms
WT C57BL/6	10^4^ TCID_50_	Weight loss up to 15% of body weight with no mortality, viremia of 8 log_10_ copies of CCHFV RNA/mL, prominent liver pathology, high inflammatory responses in the sera^19^	Weight loss up to 10% of body weight with no mortality, virus replication to titers ranging from 3–7 log copies of CCHFV RNA/mL or mg in multiple tissues at peak disease, liver pathology, inflammatory responses in the sera^19^
	10^5^ TCID_50_	Weight loss up to 25% of body weight with 80–100% mortality, viremia of 9 log_10_ copies of CCHFV RNA/mL at peak disease, severe liver pathology, high inflammatory responses including MCP-1 and TNFα in the sera, which are also associated with fatal human CCHF cases^25^	Not available
CC003	10^4^ TCID_50_	Weight loss of up to 15% of body weight and 100% mortality, viremia of 10 log_10_ copies of CCHFV RNA/mL at peak disease, severe liver pathology, high inflammatory responses MCP-1 and TNFα in the sera, which are also associated with fatal human CCHF cases	Weight loss up to 10% of body weight and no mortality, viremia of 6–7 log_10_ copies of CCHFV RNA/mL at peak disease, mild liver pathology, no inflammatory responses
CC046	10^4^ TCID_50_	Weight loss of up to 15% of body weight with 100% mortality, viremia of 10 log_10_ copies of CCHFV RNA/mL at peak disease, severe liver pathology, high inflammatory responses MCP-1 and TNFα in the sera, which are also associated with fatal human CCHF cases	Weight loss of up to 10% of body weight and 30% mortality, viremia of 6–7 log_10_ copies of CCHFV RNA/mL at peak disease, mild to moderate liver pathology, low to no inflammatory responses
CC004	10^4^ TCID_50_	Weight loss up to 17% of body weight with 45% mortality, viremia of 6–7 log_10_ copies of CCHFV RNA/mL at peak disease, liver pathology, no inflammatory responses in the sera	Weight loss up to 20% of body weight with no mortality, viremia of up to 7–9 log_10_ copies of CCHFV RNA/mL at peak disease, mild to moderate liver pathology, high inflammatory responses in the sera
CC037	10^4^ TCID_50_	Weight loss up to 11% of body weight with no mortality, viremia of 7–9 log_10_ copies of CCHFV RNA/mL at peak disease, mild liver pathology, inflammatory responses in the sera	Weight loss up to 5% of body weight with no mortality, viremia of 5 -7 log_10_ copies of CCHFV RNA/mL at peak disease, mild liver pathology, minimal inflammatory responses in the sera
CC042	10^4^ TCID_50_	No weight loss or mortality, viremia of 3–5 log_10_ copies of CCHFV RNA/mL at peak disease, no liver pathology, no inflammatory responses in the sera	No weight loss or mortality, viremia of 3–4 log_10_ copies of CCHFV RNA/mL at peak disease, no liver pathology, no inflammatory responses in the sera
CC012	10^4^ TCID_50_	No weight loss or mortality, viremia of 6–7 log_10_ copies of CCHFV RNA/mL at peak disease, minimal liver pathology, no inflammatory responses in the sera	Mild weight loss of 4% of body weight with no mortality, viremia of 6–7 log_10_ copies of CCHFV RNA/mL at peak disease, minimal liver pathology, inflammatory responses in the sera

Our study has several important limitations. First, the significant difference in disease between MA-CCHFV-infected male and female mice has not been reported in human CCHFV infections. Nevertheless, the similar correlates of disease severity in MA-CCHFV-infected mice and infected humans, along with distinct disease outcomes in a genetically variable population infected with the same strain of virus, provide a powerful tool to investigate how host genetics contribute to CCHF outcome. Second, we used MA-CCHFV which was generated by serial passage in mice on the C57BL6/J background. WT C57/BL6J mice are resistant to disease upon infection with human clinical isolates of CCHFV^19^, and therefore, we did not test exposure of the CC strains to the WT virus. Thus, it is possible that adaptation of MA-CCHFV to C57BL6/J mice may link to host genes not linked to disease in humans. Correlation of host determinants in CCHFV-infected mice will need to be confirmed in human data sets. Encouragingly, host determinants in Ebola virus disease and SARS-CoV-2 identified in CC mice were also important in human infections^36,38–40^ demonstrating the utility of the CC platform in understanding viral pathogenesis. Third, CCHFV is a genetically diverse virus, and case fatality rates can vary by region^35,41–43^. MA-CCHFV is based on CCHFV strain Hoti, isolated from a fatal human case in Eastern Europe. Therefore, we were unable to model the contribution of viral diversity to disease outcome and it is possible that distinct strains of CCHFV may interact with distinct host factors to cause disease. Mouse-adaptation or introduction of identified mouse-adaptive mutations into other strains of CCHFV will be needed to investigate the contribution of viral genetic diversity to disease outcome. Fourth, we did not perform paired timed necropsies on each strain at all time points, and thus, differences observed in pathology, cytokines, or viral loads between sexes and strains may be due to different time points analyzed. In this report we intended to establish the MA-CCHFV infection of CC mice as a model to investigate host contributions to disease outcome. Future studies will perform more in-depth temporal analyses of these factors at multiple time points.

In conclusion, using just ten CC lines, we were able to recapitulate the full spectrum of CCHF manifestations, providing strong evidence that the host genetic background is critical in determining the fate of MA-CCHFV-infected mice. Our study demonstrates the utility of the CC model for examining host responses associated with the range of disease phenotypes observed in CCHF infection. Similar correlates of disease severity measured in human CCHF cases, such as viral loads, tissue pathology, and inflammatory cytokines, were also measured in MA-CCHFV-infected CC mice. Identifying host genes that can be linked to these distinct phenotypes is the focus of ongoing studies. Using a systems biology approach, the CC can be used to assess the interaction of genetic diversity and host responses leading to disease. These studies will allow for a more thorough understanding of CCHFV pathogenesis and host responses, eventually leading to the identification of novel therapeutic interventions for CCHF.

## Supplementary information

Supplementary information

## Data Availability

All data needed to evaluate the conclusions in the paper are present in the paper and/or the Supplementary Materials.
