# Supplementary material for: Host genetic diversity contributes to disease outcome in Crimean-Congo hemorrhagic fever virus infection
Source: Npj Viruses. 2025 Feb 27;3:16. doi: 10.1038/s44298-025-00100-5 (PMC11868557; doi:10.1038/s44298-025-00100-5)
Supplement: Supplementary file 1 — Supplementary information [file 44298_2025_100_MOESM1_ESM.pdf]

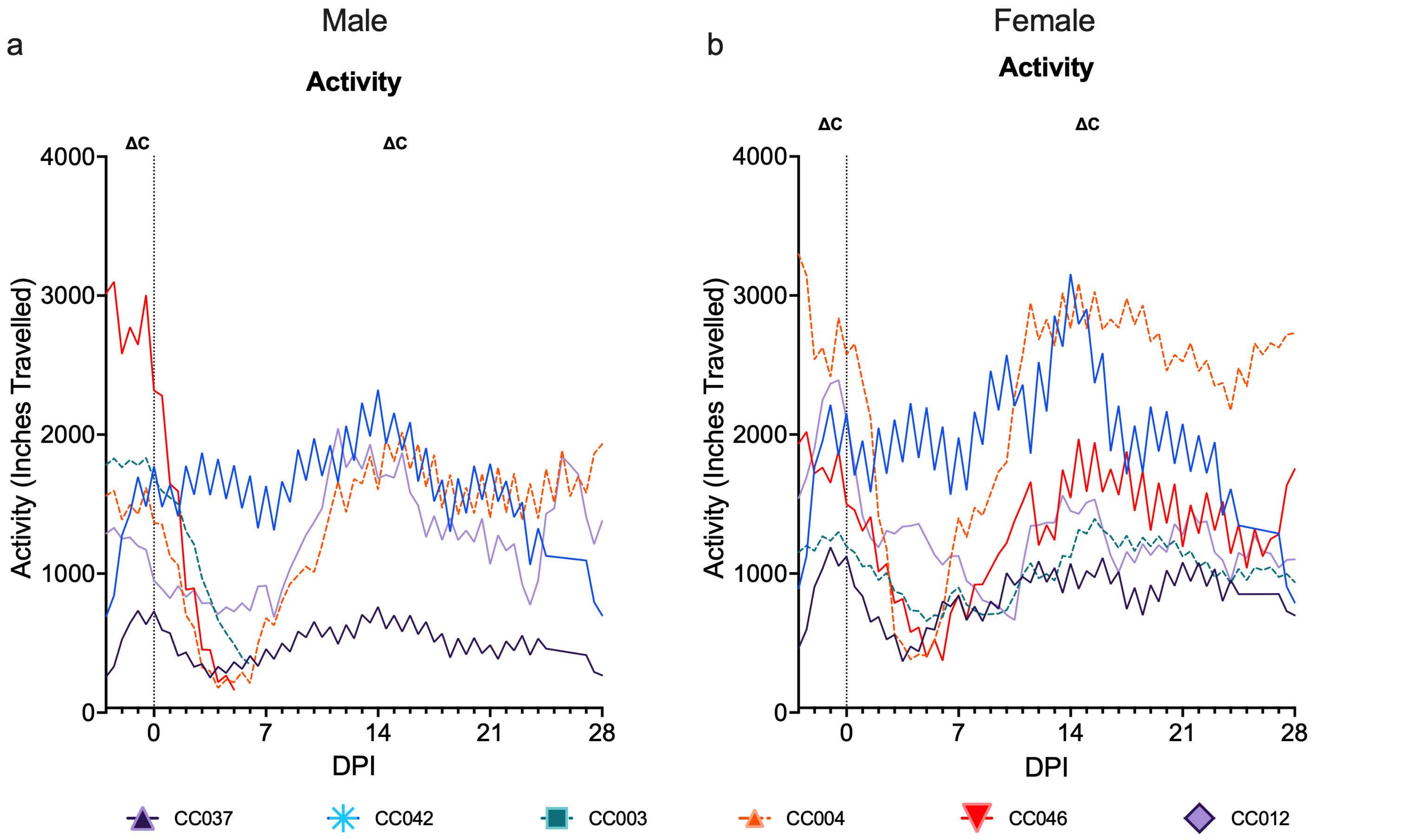

Supplemental Figure 1: Comparison of activity levels after infection of six CC mice strains with MA-CCHFV**.** Male (a – d) or female (e – h) mice of four CC strains were infected with 10^4 $TCID_{50}$ of MA-CCHFV intraperitoneally and monitored for activity levels via the telemetry system. N = 5 mice per sex for strains CC003 and CC042. N = 5 males and 3 females for strain CC037. N = 9 mice per sex for strain CC004. N = 10 mice per sex for strains CC046 and CC012. Activity of male (a) and female (b) mice was measured by the cage telemetry system. Data points were smoothed using 4 neighbors to each side and a 2nd order polynomial. Cages were changed on days -1 and 14 relative to infection, indicated by ΔC.

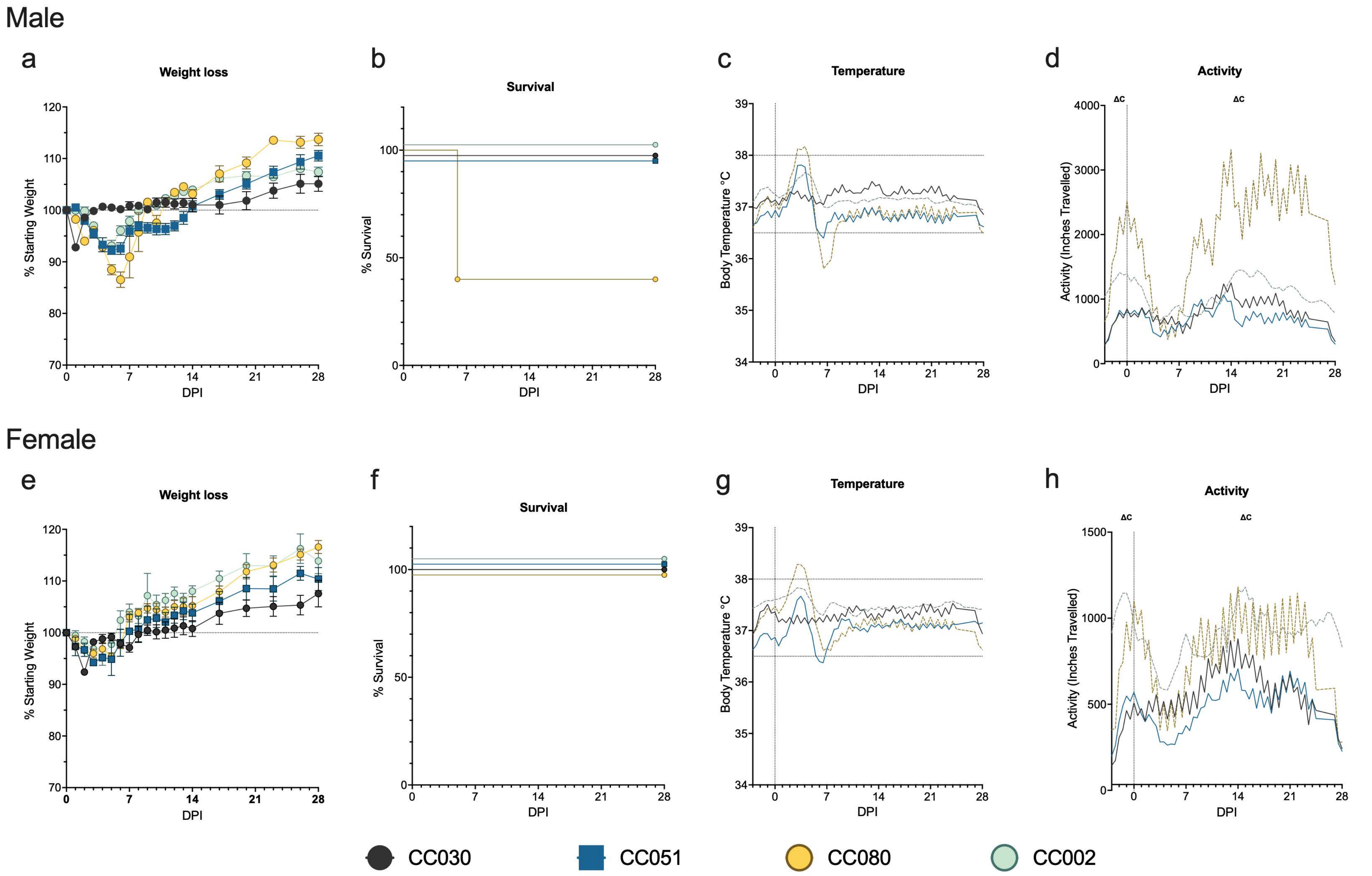

Supplemental Figure 2: Comparison of disease parameters after infection of four CC mice strains with MA-CCHFV**.** Male (a – d) or female (e – h) mice of four CC strains were infected with 10^4 $TCID_{50}$ of MA-CCHFV intraperitoneally and monitored for clinical signs and symptoms of disease. N= 5 mice per sex per strain for all strains. Mice were weighed daily, (a & e), monitored for survival (b & f) and body temperature (c & g) and activity (d & h) was measured by the cage telemetry system. Data shown as mean plus SEM (a & e). For telemetry data (c, d, g, h) data points were smoothed using 4 neighbors to each side and a 2nd order polynomial. Cages were changed on days -1 and 14 relative to infection, indicated by ΔC (d & h).

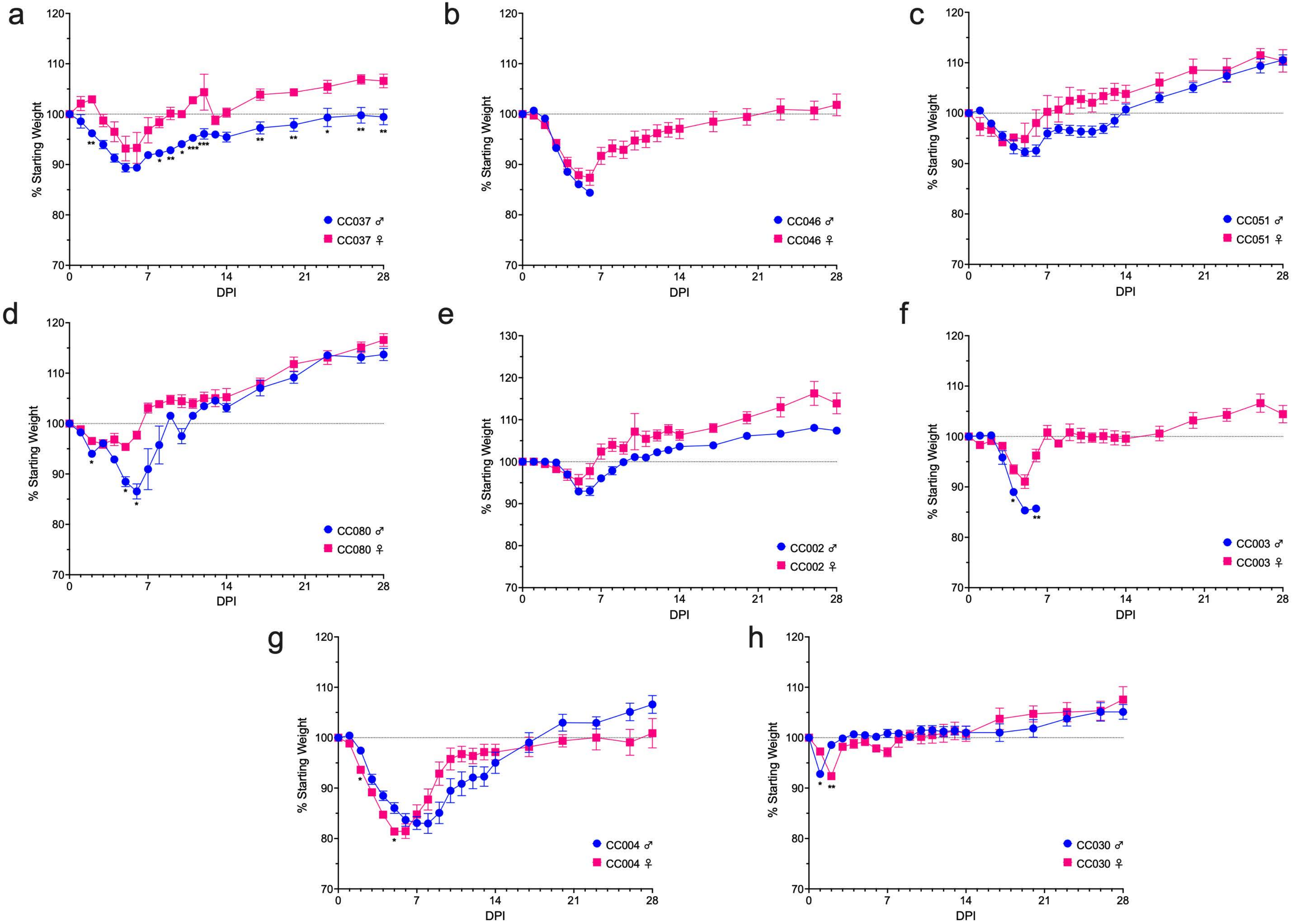

Supplemental Figure 3: Weight loss comparisons between male and female mice of each strain of CC mice susceptible to symptomatic disease after infection with MA-CCHFV. Male and female mice of each CC strain were infected with MA-CCHFV and monitored for weight loss. N = 5 mice per sex for strains CC003, CC042, CC030, CC051, CC080 and CC002. N = 5 males and 3 females for strain CC037. N = 9 mice per sex for strain CC004. N = 10 mice per sex for strains CC046 and CC012. Data from two independent experiments combined for strains CC046, CC012 and CC004. Statistics were calculated with a two-way ANOVA or mixed-effect analysis with Sidak's multiple comparison test for weight loss comparisons. *P < 0.05, **P < 0.01, ***P < 0.001, ****P < 0.0001.

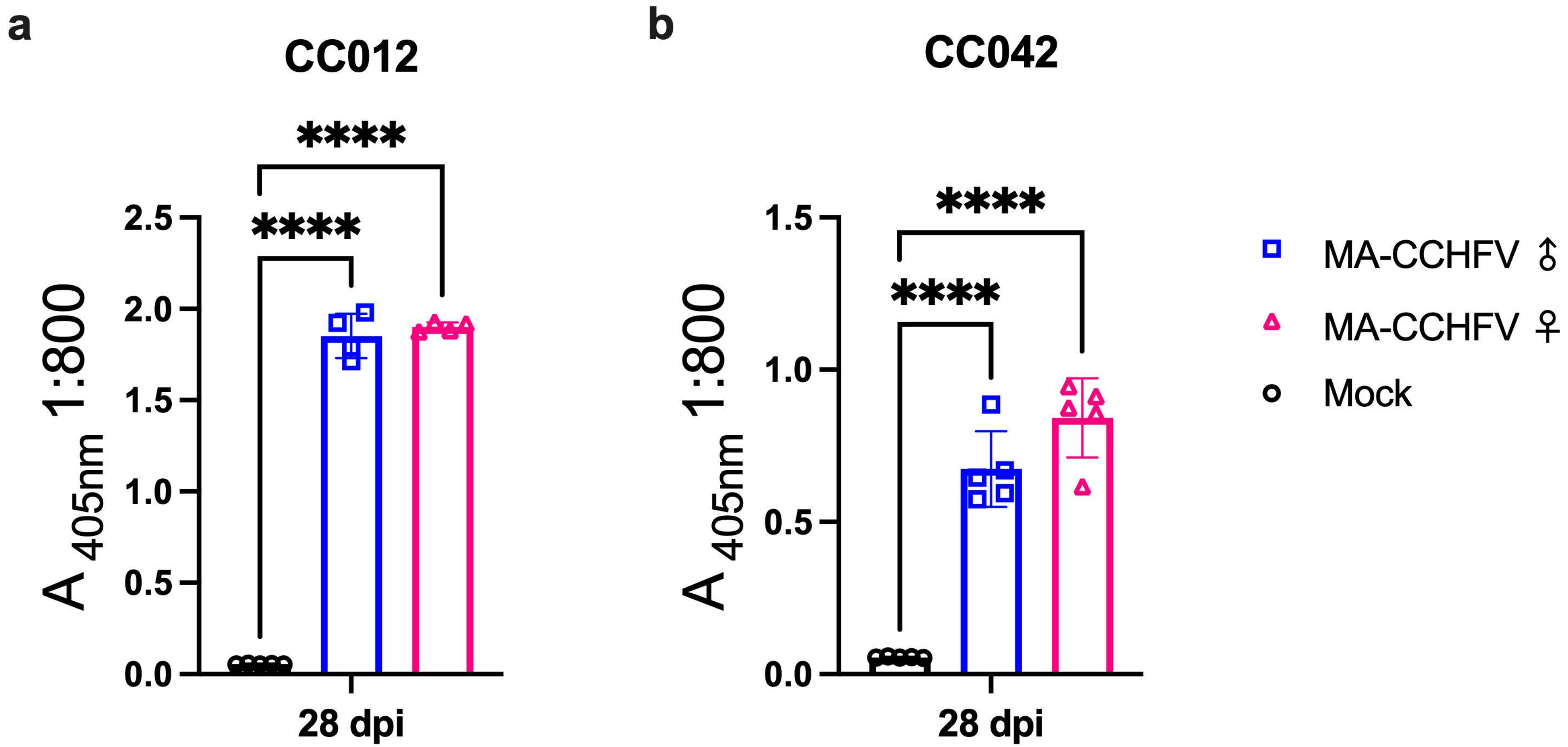

Supplemental Figure 4: IgG responses to CCHFV in the sera of MA-CCHFV infected male and female mice of strains CC012 and CC042. Sera from male and female mice of strains CC012 and CC042 infected with MA-CCHFV were analyzed at 28 days post-infection for CCHFV-specific IgG responses by ELISA compared to mock sera. N = 4 mice per sex for strain CC012J and N = 5 mice per sex for strain CC042. Statistics were calculated with a one-way ANOVA with Tukey's multiple comparison test. $^{*}P < 0.05$, $^{**}P < 0.01$, $^{***}P < 0.001$, $^{****}P < 0.0001$.

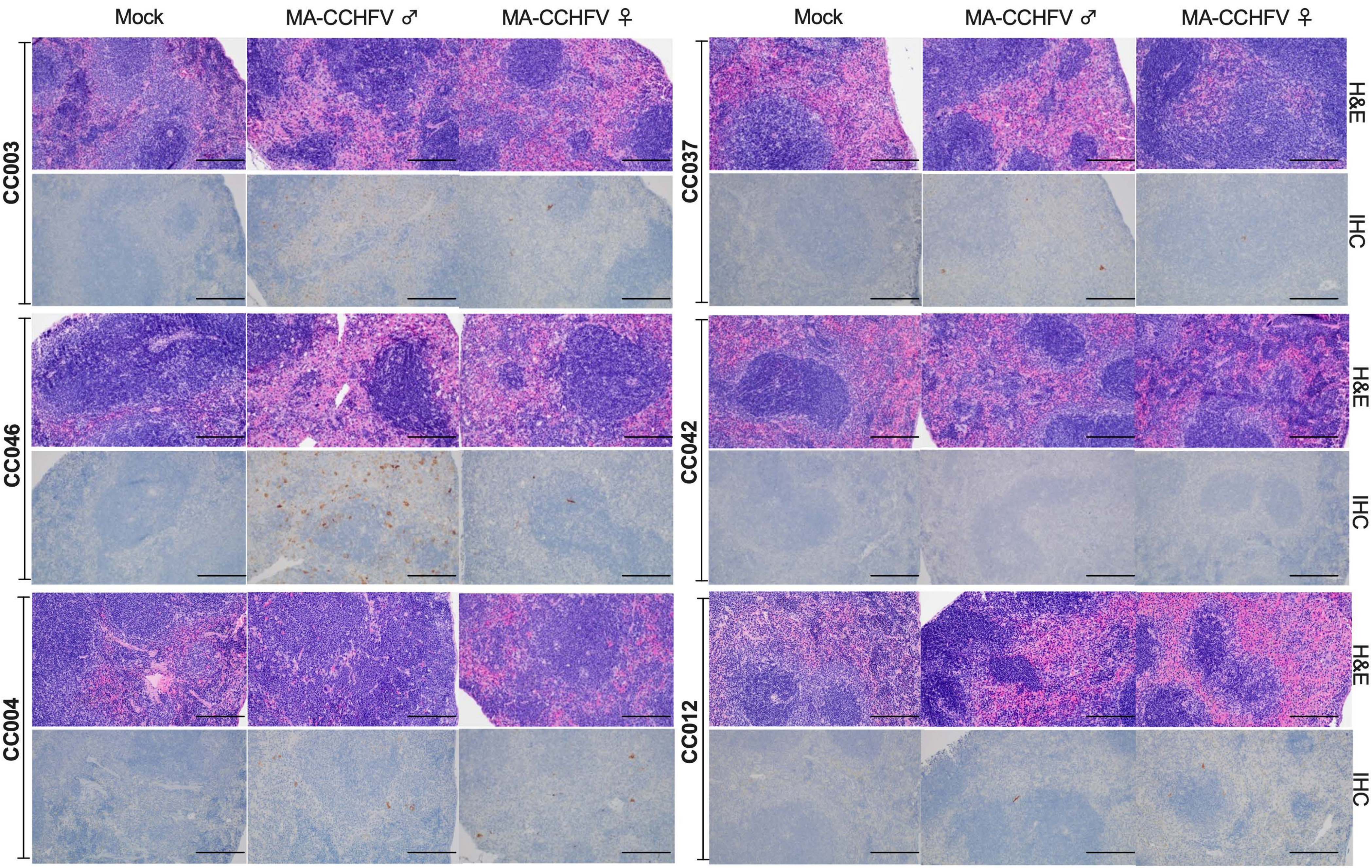

Supplemental Figure 5: Pathology and anti-CCHFV immunoreactivity in spleens of male and female mice of each strain of CC mice after infection with MA-CCHFV. Male and female mice of 6 CC strains were infected with MA-CCHFV and necropsied at peak disease. Spleen sections were analyzed for pathology by H&E staining and for CCHFV antigen by anti-NP IHC.

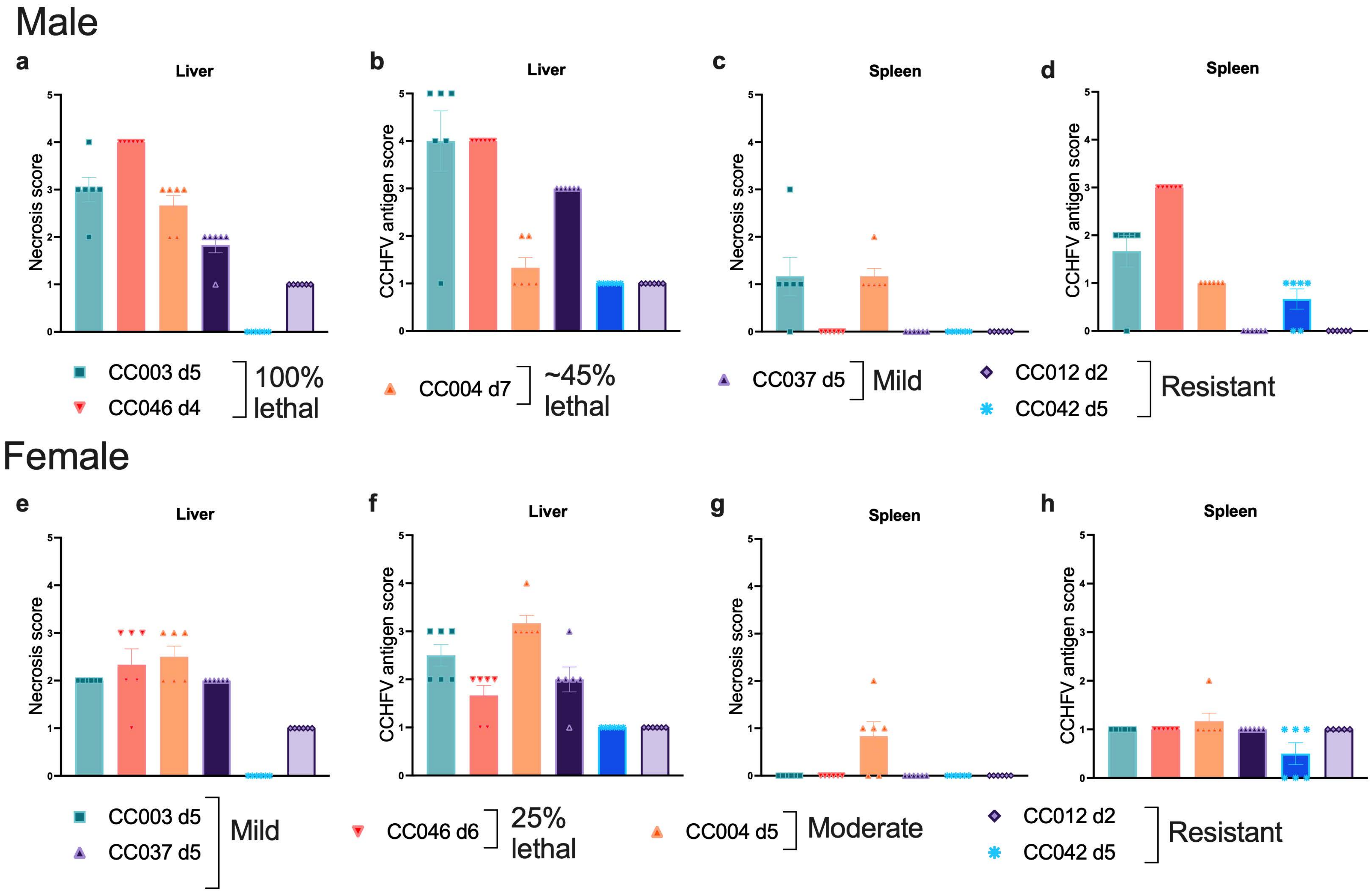

Supplemental Figure 6: Pathology scores in livers and anti-CCHFV immunoreactivity scores in livers and spleens of male and female mice of the 6 strains of CC mice after infection with MA-CCHFV. Male and female mice of 6 CC strains were infected with MA-CCHFV and necropsied at peak disease. Liver and spleen sections processed for pathology and CCHFV antigen by IHC were scored for necrosis and CCHFV antigen on a scale of 1-5.

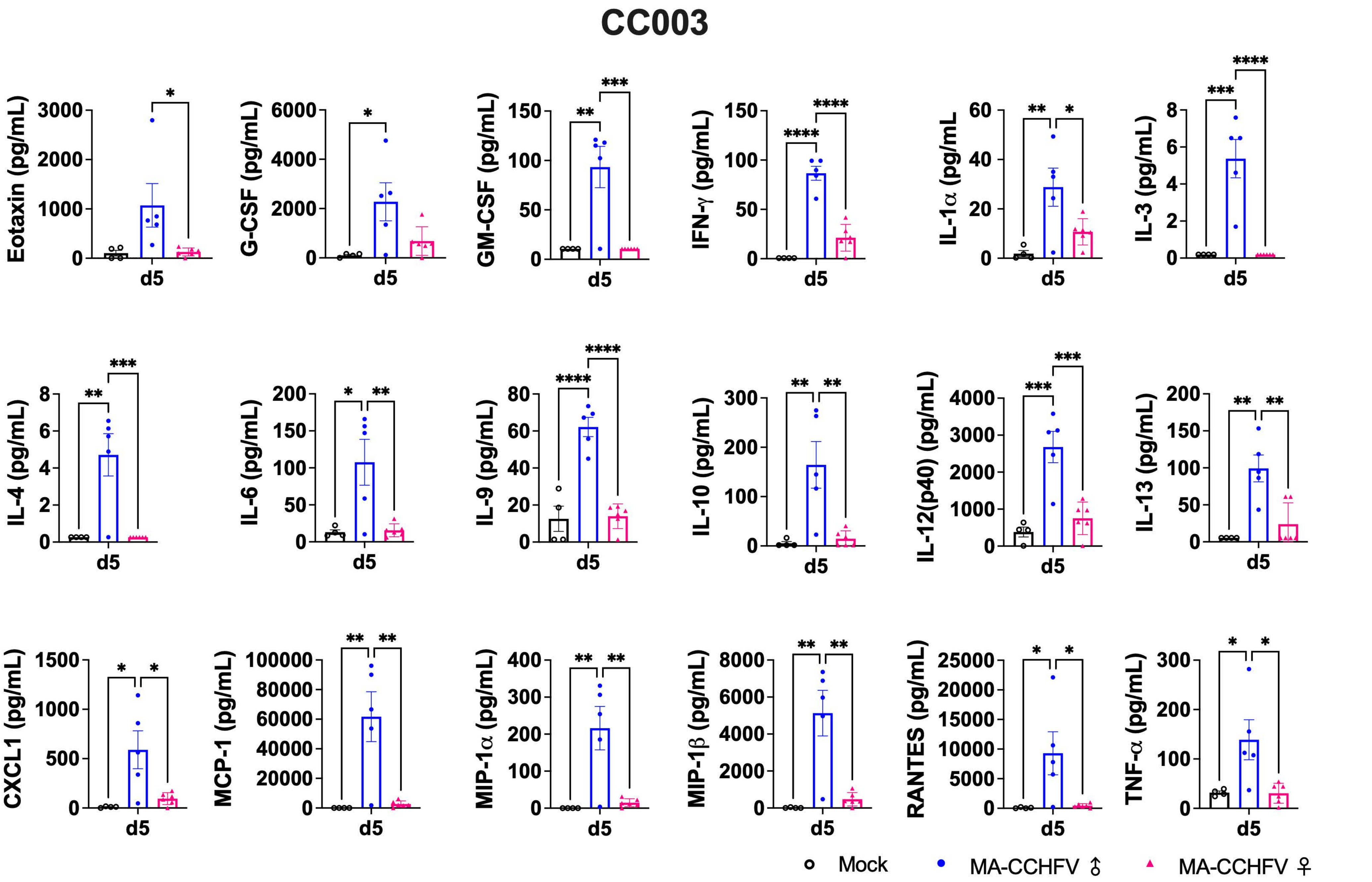

Supplemental Figure 7: Inflammatory responses to CCHFV in the sera of MA-CCHFV infected male and female mice of strain CC003. Sera from infected male and female mice at day 5 post-infection were analyzed for inflammatory cytokine levels compared to mock sera. N = 5 male mice and N = 6 female mice. Statistics were calculated with a one-way ANOVA with Tukey's multiple comparison test. *P < 0.05, **P < 0.01, ***P < 0.001, ****P < 0.0001.

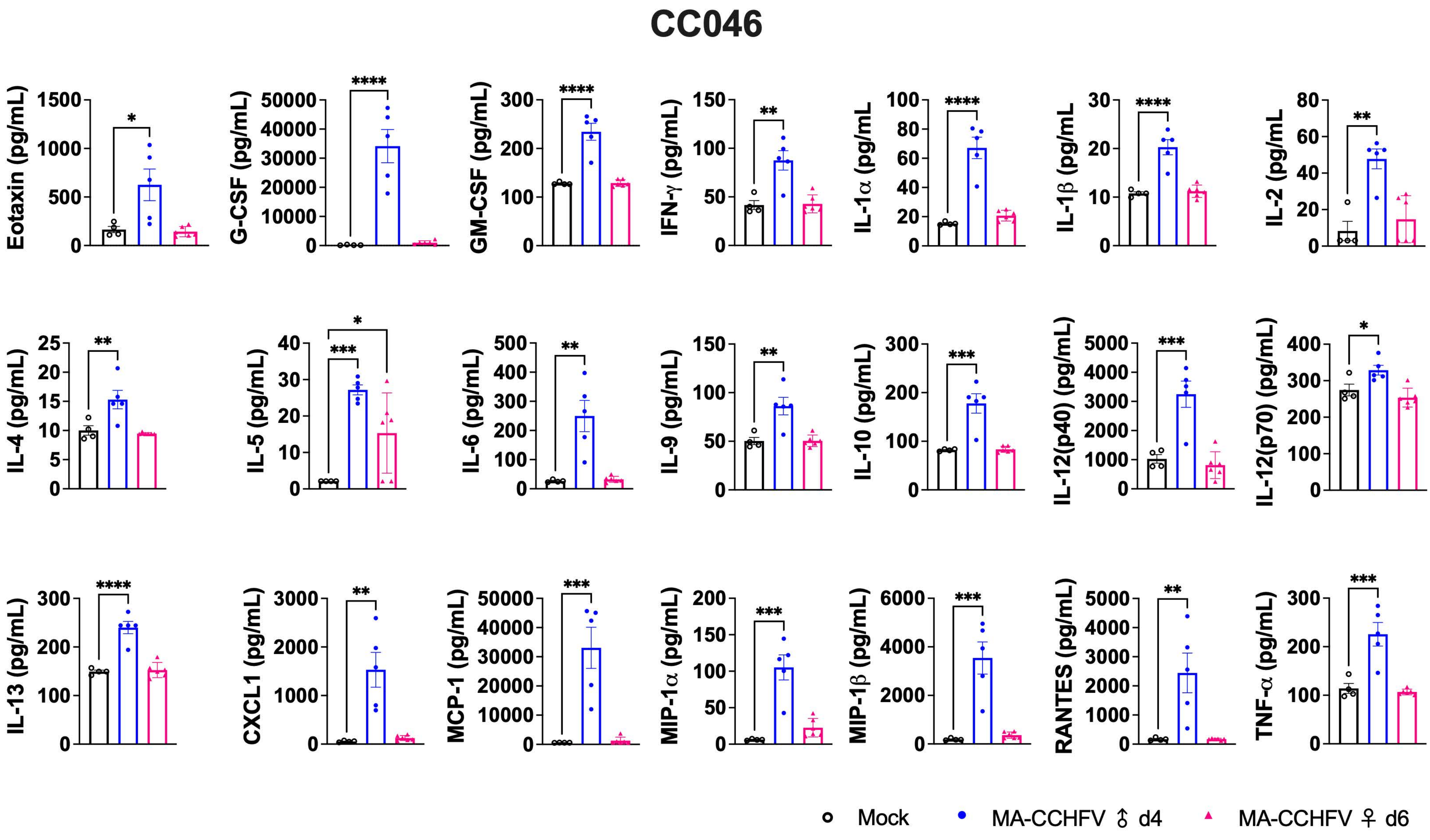

Supplemental Figure 8: Inflammatory responses to CCHFV in the sera of MA-CCHFV infected male and female mice of strain CC046. Sera from infected male mice at day 4 and female mice at day 6 post-infection were analyzed for inflammatory cytokine levels compared to mock sera. N = 5 male mice and N = 6 female mice. Statistics were calculated with a one-way ANOVA with Tukey's multiple comparison test. *P < 0.05, **P < 0.01, ***P < 0.001, ****P < 0.0001.

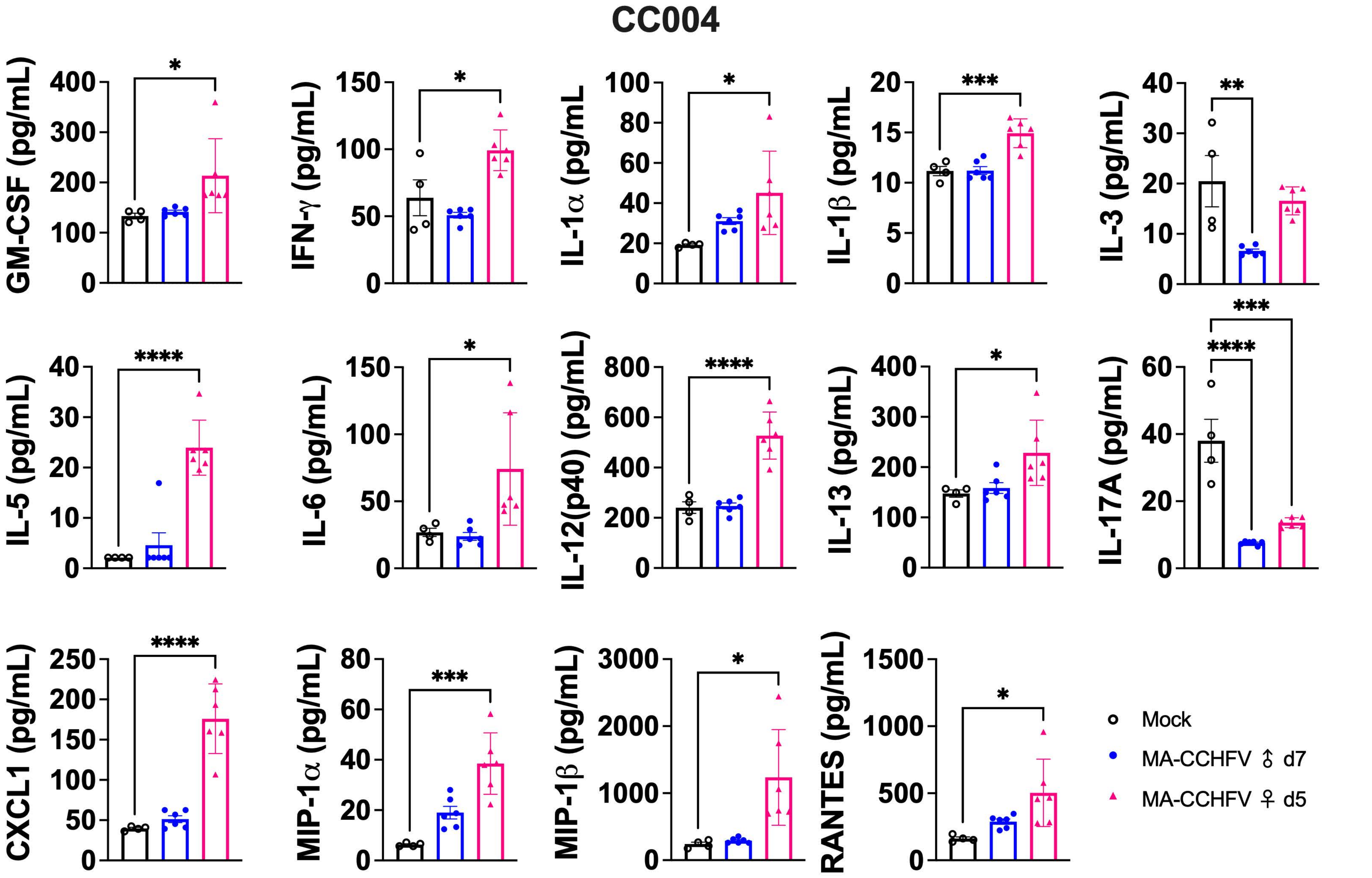

Supplemental Figure 9: Inflammatory responses to CCHFV in the sera of MA-CCHFV infected male and female mice of strain CC004. Sera from infected male mice at day 7 and female mice at day 5 post-infection were analyzed for inflammatory cytokine levels compared to mock sera. N = 6 mice per sex. Statistics were calculated with a one-way ANOVA with Tukey's multiple comparison test. *$P < 0.05$, **$P < 0.01$, ***$P < 0.001$, ****$P < 0.0001$.

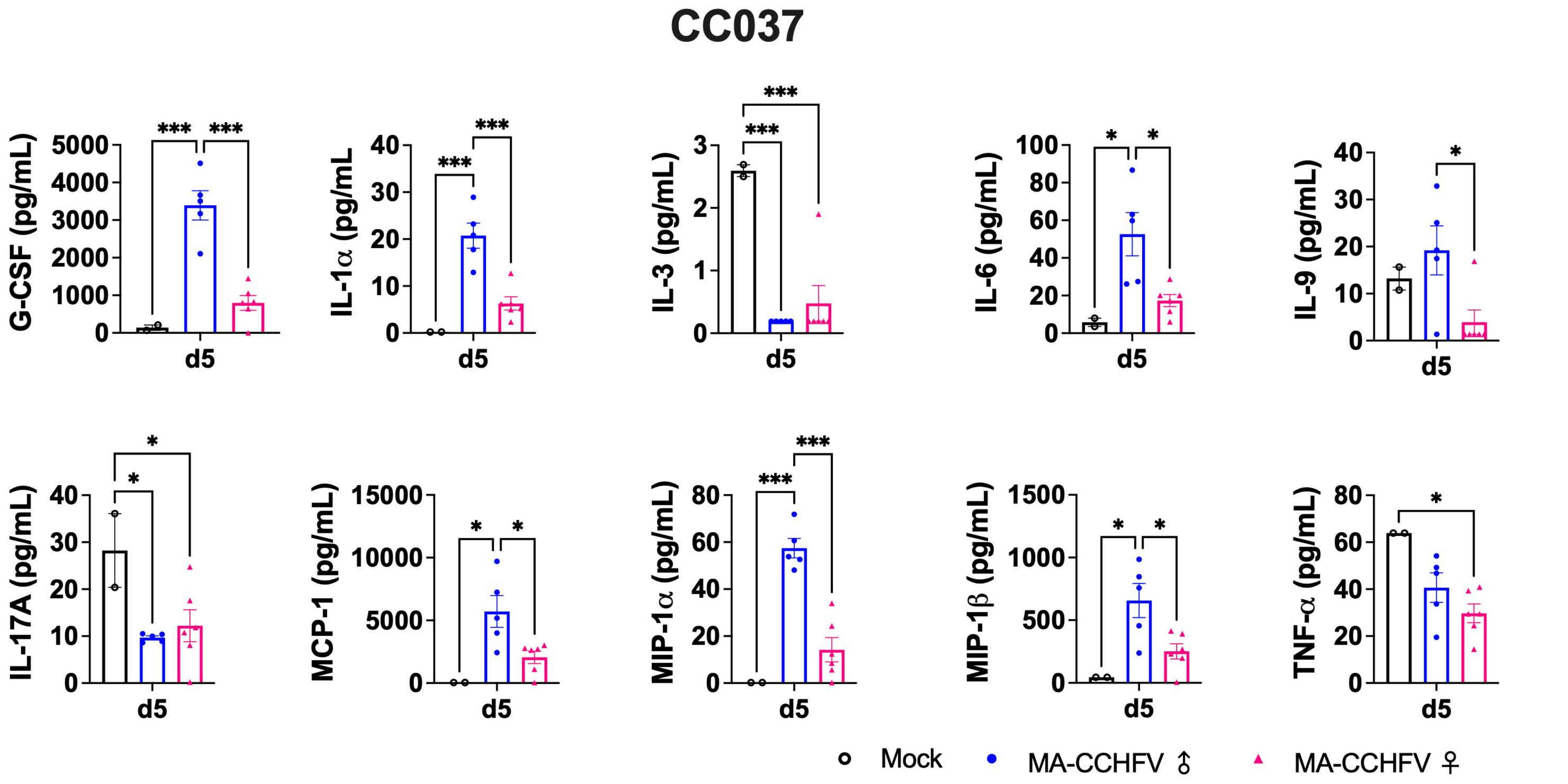

Supplemental Figure 10: Inflammatory responses to CCHFV in the sera of MA-CCHFV infected male and female mice of strain CC037. Sera from infected male and female mice at day 5 post-infection were analyzed for inflammatory cytokine levels compared to mock sera. N = 5 male mice and N = 6 female mice. Statistics were calculated with a one-way ANOVA with Tukey's multiple comparison test. *P < 0.05, **P < 0.01, ***P < 0.001, ****P < 0.0001.

CC042

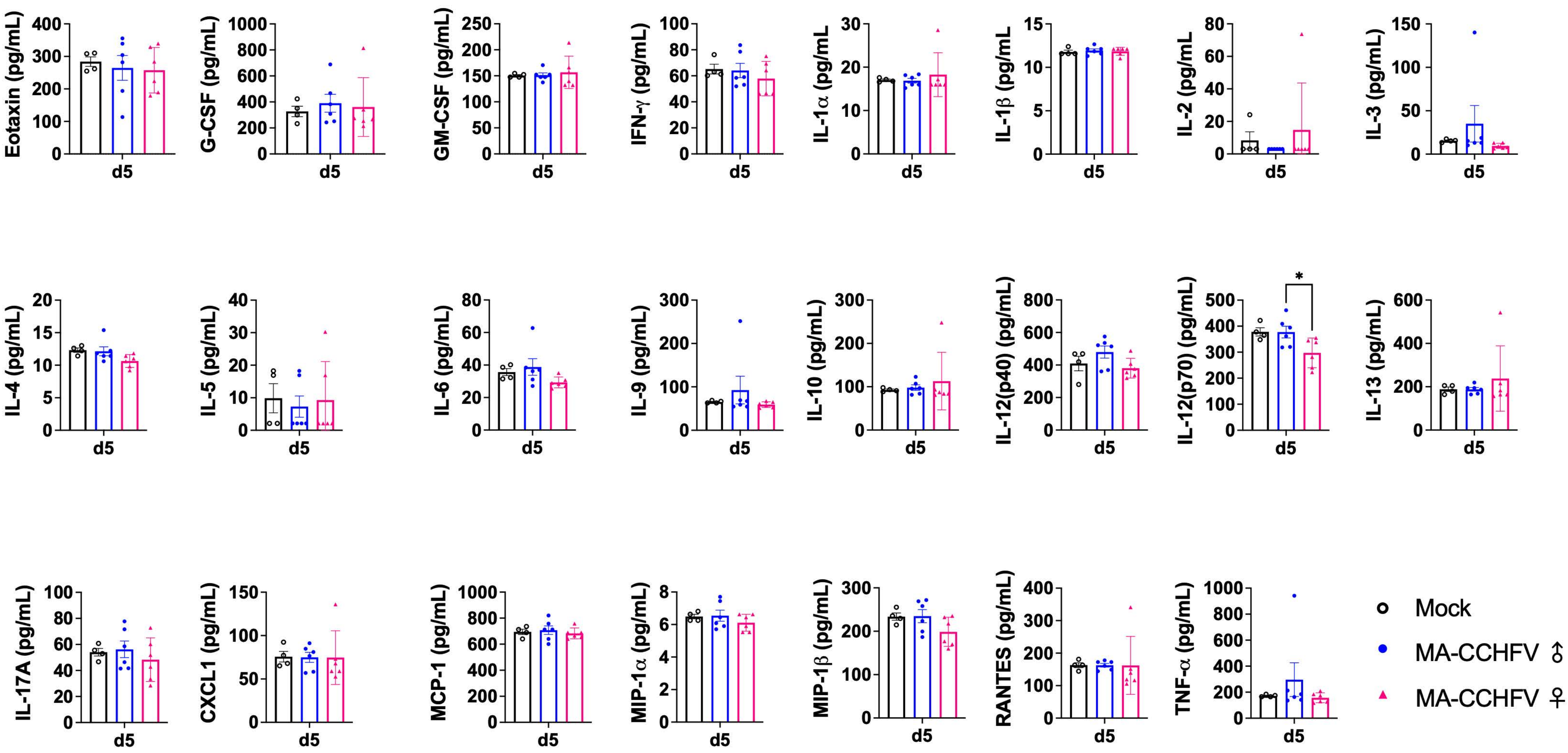

Supplemental Figure 11: Inflammatory responses to CCHFV in the sera of MA-CCHFV infected male and female mice of strain CC042. Sera from infected male and female mice at day 5 post-infection were analyzed for inflammatory cytokine levels compared to mock sera. N = 6 mice per sex. Statistics were calculated with a one-way ANOVA with Tukey's multiple comparison test. *P < 0.05, **P < 0.01, ***P < 0.001, ****P < 0.0001.

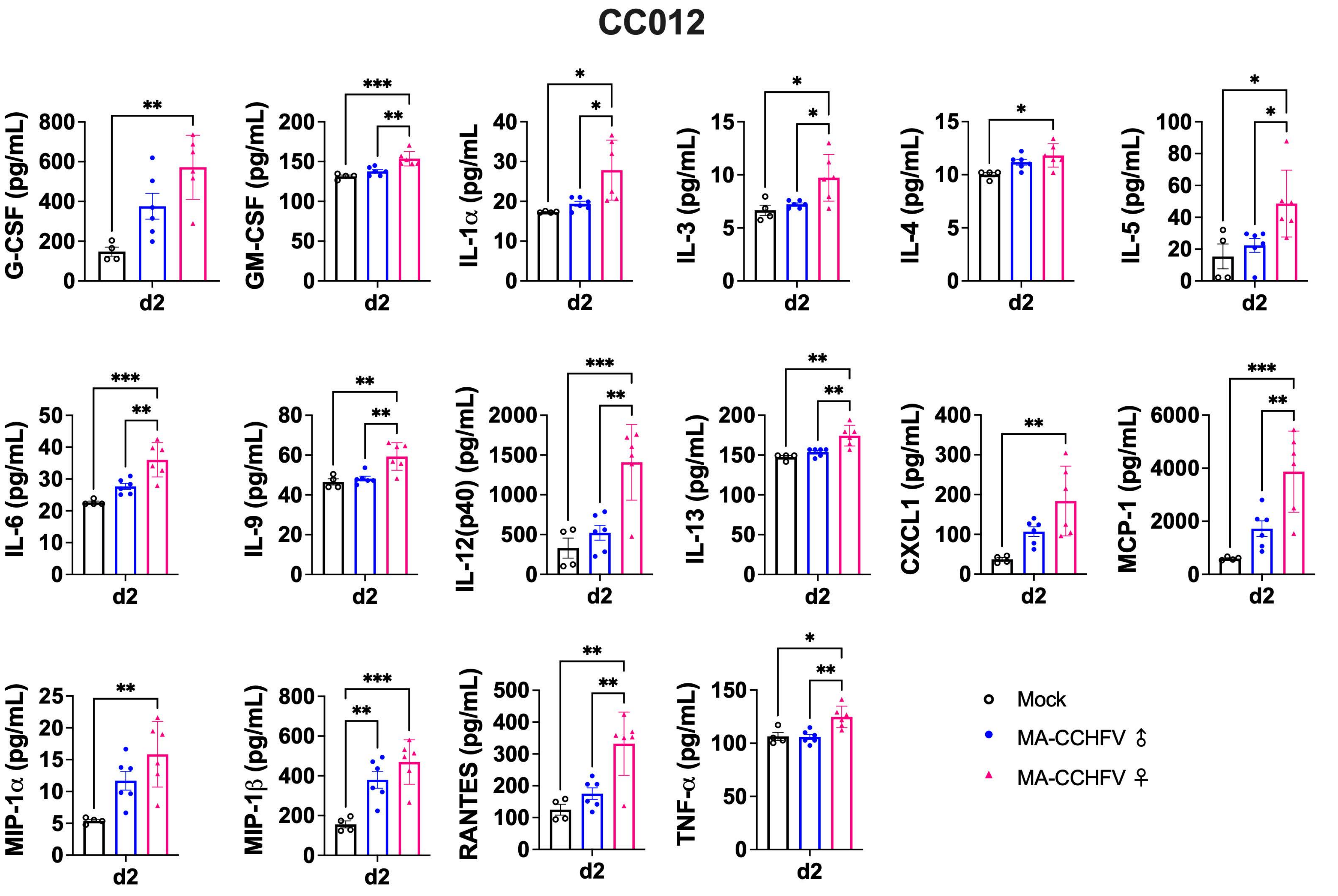

Supplemental Figure 12: Inflammatory responses to CCHFV in the sera of MA-CCHFV infected male and female mice of strain CC012. Sera from infected male and female mice at day 2 post-infection were analyzed for inflammatory cytokine levels compared to mock sera. N = 6 mice per sex. Statistics were calculated with a one-way ANOVA with Tukey's multiple comparison test. *P < 0.05, **P < 0.01, ***P < 0.001, ****P < 0.0001.
